# Downregulation of ROR2 promotes dental pulp stem cell senescence by inhibiting STK4‐FOXO1/SMS1 axis in sphingomyelin biosynthesis

**DOI:** 10.1111/acel.13430

**Published:** 2021-07-18

**Authors:** Xing‐yue Dong, Yan‐xia Huang, Zhan Yang, Xiao‐yang Chu, Jue Wu, Shan Wang, Xin He, Chun‐Yan Gao, Xu Chen, Kai Yang, Dong‐liang Zhang

**Affiliations:** ^1^ Department of Orthodontics, Stomatological Hospital, Capital Medical University; Capital Medical University of Stomatology Beijing China; ^2^ Molecular Biology Laboratory, Talent and Academic Exchange Center The Second Hospital of Hebei Medical University Shijiazhang China; ^3^ Department of Stomatology Fifth Medical Center of Chinese, PLA General Hospital Beijing China; ^4^ Translational Medical Research Center Medical Innovation Research Division of Chinese PLA General Hospital Beijing China; ^5^ Prenatal Diagnosis Center Beijing Obstetrics and Gynecology Hospital Capital Medical University Beijing China

**Keywords:** dental pulp stem cells, FOXO1, ROR2, senescence, SMS1

## Abstract

Dental pulp stem cells (DPSCs) play a vital role in tooth restoration, regeneration, and homeostasis. The link between DPSC senescence and tooth aging has been well‐recognized. ROR2 plays an important role in aging‐related gene expression. However, the expression and function of ROR2 in DPSC aging remain largely unknown. In this study, we found that ROR2 expression was significantly decreased in aged pulp tissues and DPSCs. The depletion of ROR2 in young DPSCs inhibits their self‐renewal capacity, while its overexpression in aged DPSCs restores their self‐renewal capacity. Interestingly, we found that sphingomyelin (SM) is involved in the senescence of DPSCs regulated by ROR2. Mechanistically, we confirmed that ROR2 inhibited the phosphorylation of STK4, which promoted the translocation of Forkhead Box O1 (FOXO1) to the nucleus. STK4 inhibition or knockdown of FOXO1 markedly increased the proliferation of DPSCs and upregulated the expression of SMS1, which catalyzed SM biogenesis. Moreover, FOXO1 directly bound to the SMS1 promoter, repressing its transcription. Our findings demonstrated the critical role of the ROR2/STK4‐FOXO1/SMS1 axis in the regulation of SM biogenesis and DPSC senescence, providing a novel target for antagonizing tooth aging.

## Graphical abstract

Proposed hypothesis for ROR2 suppressing ageing process by promoting sphingomyelin synthesis through inactivating STK4/FOXO1 signallingin in DPSCs. ROR2 decreases with age, and overexpression of ROR2 restores the differentiation and regeneration of senescent DPSC. ROR2 prevents the translocation of FOXO1 to the nucleus by inhibiting STK4 phosphorylation. FOXO1 binds to the promoter of SMS1, which catalyzes SM biogenesis, and represses its transcription. The ROR2/STK4‐FOXO1/SMS1 axis regulated SM biogenesis and DPSC senescence.

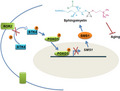

**Abbreviations**
DPSCs: Dental Pulp Stem Cells
FOXO1: Forkhead Box O1
MSCs: Mesenchymal Stem Cells
PBS: Phosphate Buffered Saline
ROR: Receptor tyrosine kinase‐like orphan receptors
ROR2: Receptor tyrosine kinase‐like orphan receptors 2
SMS1: Sphingomyelin synthetases 1
STK4: Serine/threonine‐protein kinase 4

## 1 INTRODUCTION

Dental pulp stem cells (DPSCs) are the most important type of dental stem cells that play a vital role in tooth restoration, regeneration, and homeostasis (Macrin et al., 2019; Tsutsui, 2020). Although DPSCs can be isolated and cultured from a few dental pulp samples, research and clinical applications require a large number of cells, so it is essential to expand their population by in vitro cell culture. However, under standard culture conditions, cells enter cell‐like senescence due to the limited number of divisions (Estrada et al., 2013). DPSC senescence directly affects the differentiation ability of cells, which is also an important shortcoming of stem cell therapy (Morsczeck et al., 2018). Cell senescence is a complex process; the molecular mechanism that leads to the senescence of DPSCs is still unclear.

Receptor tyrosine kinase‐like orphan receptors (ROR), including ROR1 and ROR2, the receptors of Wnt5a, play an important regulatory role by activating the Wnt/β‐catenin non‐canonical signal pathway (Dave et al., 2019; Yu et al., 2016). Studies have confirmed that ROR2 plays a vital role in regulating cell polarity, migration, proliferation, differentiation, developmental morphogenesis, and tissue/organogenesis (Debebe & Rathmell, 2015). Our previous study found a novel variation in the ROR2 gene, which causes autosomal recessive Robinow syndrome (Yang et al., 2020). A recent study reported that ROR2 is one of the important markers of human multifunctional mesenchymal stem cells (MSCs), and a high expression of ROR2 indicates that MSCs have an enhanced ability to form cartilages (Dickinson et al., 2017). ROR2 is expressed in neural stem/progenitor cells (NPC) and participates in the regulation of its proliferation (Zhang et al., 2019). The suppressed ROR2 expression in bFGF‐stimulated NIH/3T3 cells results in an increased expression of senescence‐associated genes, such as p21^Cip1^ (Endo et al., 2020). However, the role of ROR2 in the aging process in DPSCs is largely unknown.

Sphingomyelin (SM) is the major species of sphingolipids and almost 65% of cell membrane‐associated SM is rich in lipid rafts (Wang et al., 2019). SM synthetases, SMS1 and SMS2 (encoded by the SGMS1 and SGMS2 genes), are the key synthetases for the biogenesis of SM (Matsumoto et al., 2019). SMS1 is responsible for SM biogenesis in the Golgi apparatus, whereas, SMS2 is located in both the plasma membrane and the Golgi apparatus (Villani et al., 2008; Yeang et al., 2008). Studies have reported that SMS1‐mutant mice exhibit moderate neonatal lethality, reduced body weight, loss of fat‐tissue mass, and growth deterioration (Yano et al., 2011). The majority of studies that associated SMS1 gene expression with cancer development had aimed to determine SM levels or SMS enzyme activity (Yeang et al., 2008; Yu et al., 2016; Zhang et al., 2019). CD4+ T cells from SMS1 (‐/‐) mice showed severe deficiency in membrane SM and inhibited cellular proliferation (Dong et al., 2012). A recent study reported that SM was significantly decreased in old mouse kidneys and was associated with aging (Noh et al., 2019). However, the role of SMS1 in the aging process of DPSCs is still unclear.

In this study, we investigated the ROR2 expression in pulp tissues and DPSCs isolated from donors at different ages. We demonstrated that ROR2 expression in DPSCs declines with age, and ROR2 plays a vital role in the senescence of DPSCs. The overexpression of ROR2 in aged DPSCs restores their self‐renewal capacity. Additionally, we found that SM is involved in the senescence of DPSCs regulated by ROR2. Furthermore, the STK4‐FOXO1 axis mediates ROR2 to regulate the expression of SMS1, which directly regulates the SM biogenesis in DPSCs. Our results indicated that ROR2 could be an ideal target for antagonizing DPSC aging.

## 2 RESULTS

### 2.1 The expression of ROR2 was downregulated in senescent DPSCs

**FIGURE 1:**
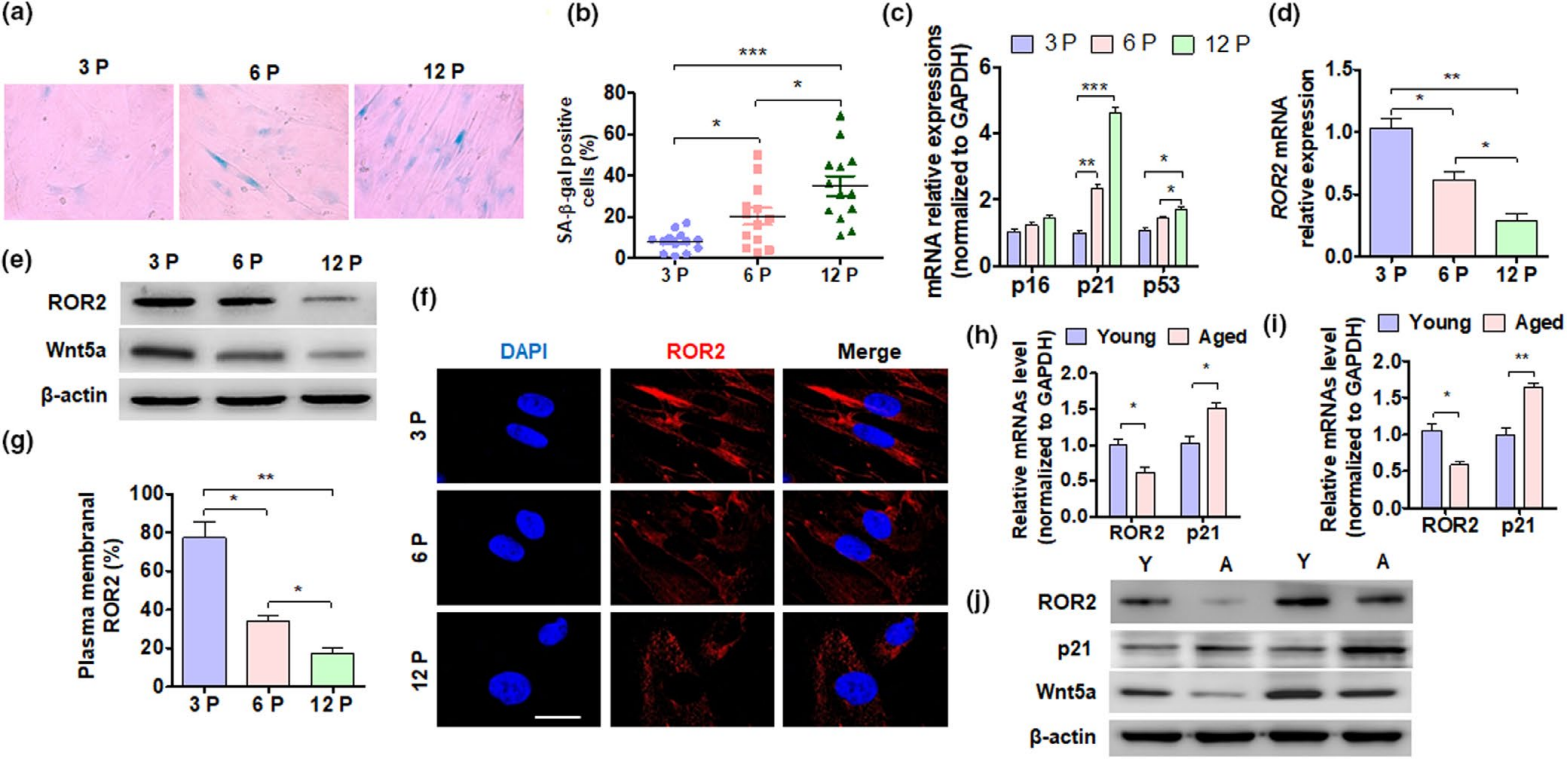
ROR2 expression is reduced in aged DPSCs. (a) DPSC aging was induced by cell passage. SA β‐gal staining of DPSCs from young donors at passage 3 (3P), 6 (6P), and 12 (12P). (b) Quantitative analysis of SA β‐gal‐positive DPSCs (3P, 6P, and 12P). (c and d) RT‐qPCR was performed to detect the p16, p21, p53, and ROR2 mRNA expression in DPSCs (3P, 6P, and 12P). (e) Western blot analysis was performed to detect the ROR2 protein level in DPSCs from young donors. (f) Immunofluorescence staining for ROR2 in DPSCs; scale bar: 20 µm. (g) Quantitative analysis of cells with ROR2 in the plasma membrane of (f). (h and i) RT‐qPCR was performed to detect the ROR2 and p21 mRNA expression in the dental pulp tissues of young and elderly patients. (h) DPSC isolation (6P) from young and aged donors. (j) Western blot analysis of ROR2 and p21 expression in DPSCs (6P) isolated from young (Y) and aged (A) donors. For all analyses, *p < 0.05, **p < 0.01, and ***p < 0.001 compared to their corresponding controls

Previous studies had reported that MSCs cultured to passage six would have a senescence phenotype (Ma et al., 2019). First, we induced the senescence of DPSCs by cell passaging, and SA β‐gal staining was performed to detect cell senescence. The results showed that the number of SA β‐gal‐positive cells increased at passage six, and this further elevated at passage twelve (Figure 1a,b). The proliferation of DPSCs decreased at passage six and reduced further at passage twelve (Figure S1). Flow cytometry data indicated that the DPSCs expressed mesenchymal stem cell‐associated surface markers, such as CD44, CD73, and CD105; neither cell type expressed CD34 or CD45 (Table S1). Results of the RT‐qPCR also confirmed that DPSCs at passage twelve had much higher expression of senescence marker genes, such as p21 and p53 (Figure1c). A recent study had reported that the knockdown of ROR2 lead to the upregulation of cell senescence marker genes, such as p21 and the cell cycle resistance cyclin‐dependent kinase inhibitor 1B (p27) (Endo et al., 2020). We determined whether ROR2 and its ligand Wnt5a were involved in the senescence of DPSCs by performing cell passaging. As expected, our result showed that the expression of ROR2 and Wnt5a was significantly downregulated with the increase in the passage number of cells (Figure 1d,e, Figure S2a). Additionally, immunofluorescence staining also confirmed that the level of ROR2 protein markedly decreased as the passage number of DPSCs increased (Figure 1f). Furthermore, we detected the ROR2 and Wnt5a expression in human dental pulp tissues and DPSCs from donors. Our results showed that the expression of ROR2 and Wnt5a was higher in the tissues and DPSCs from young donors than that from old donors, while the expression of p21 showed the opposite pattern (Figure 1h‐j, Figure S2b). These results indicate that the expression of ROR2 was reduced in senescent DPSCs.

### 2.2 ROR2 regulates the senescence of DPSCs

**FIGURE 2:**
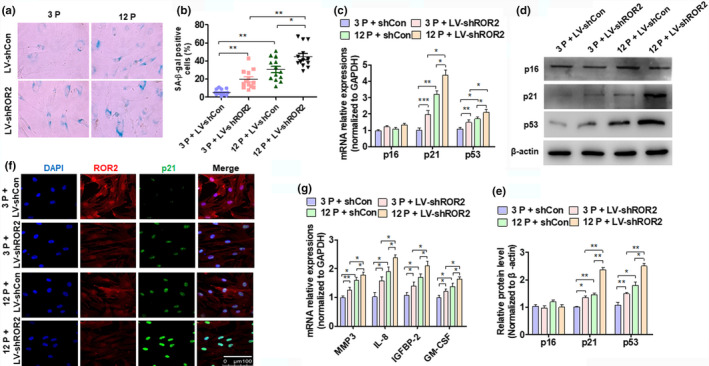
ROR2 regulates DPSC senescence. (a) DPSCs at passage 3 (3P) and 12 (12P) from young donors were transfected with LV‐shCon and LV‐shROR2, followed by staining with SA β‐gal. (b) Quantitative analysis of the transfected SA β‐gal‐positive DPSCs (3P and 12P). (c) RT‐qPCR was performed to detect the expression of p16, p21, and p53 mRNA in the transfected DPSCs (3P and 12P). (d) DPSC (3P and 12P) treatment as (c) and Western blot analysis was performed to detect the expression of p16, p21, and p53 protein levels. (e) Quantitative analysis of (D). (f) Immunofluorescence staining for ROR2 and p21 in the transfected DPSCs (3P and 12P); scale bar: 100 µm. (g) RT‐qPCR for SASP gene (MMP3, IL‐8, IGFBP‐2, and GM‐CSF) expressions in the transfected DPSCs (3P and 12P). For all analyses, *p < 0.05, **p < 0.01, and ***p < 0.001 compared to their corresponding controls

To investigate whether ROR2 was involved in cell senescence, we knocked down the expression of ROR2 in 3P and 12P cells and used SA β‐gal stain to detect DPSC senescence. As shown in Figure 2a,b, the quantitative analysis indicated that SA β‐gal‐positive DPSCs had significantly increased in the 3P and 12P DPSCs after ROR2 knockdown. The proliferation of DPSC significantly decreased in ROR2‐depleted cells (Figure S3) Moreover, the expression of senescent marker genes p21 and p53 was markedly elevated in both mRNA and protein levels in the ROR2‐depleted DPSCs (Figure 2c–e). Furthermore, immunofluorescence staining also confirmed that ROR2 knockdown promoted the expression of p21 in DPSCs (Figure 2f). Additionally, we tested the expression of senescence‐associated secretory phenotype (SASP) genes, including the expression of MMP3, IL‐8, IGFBP‐2, and GM‐CSF, and found that these SASP genes were highly upregulated in the DPSCs from the elderly; knocking down ROR2 further increased the expression of the SASP genes (Figure 2g). These results suggested that the depletion of ROR2 promoted DPSC senescence.

### 2.3 ROR2 depletion in young DPSCs inhibits, while ROR2 overexpression in aged DPSCs restores self‐renewal capacity

**FIGURE 3:**
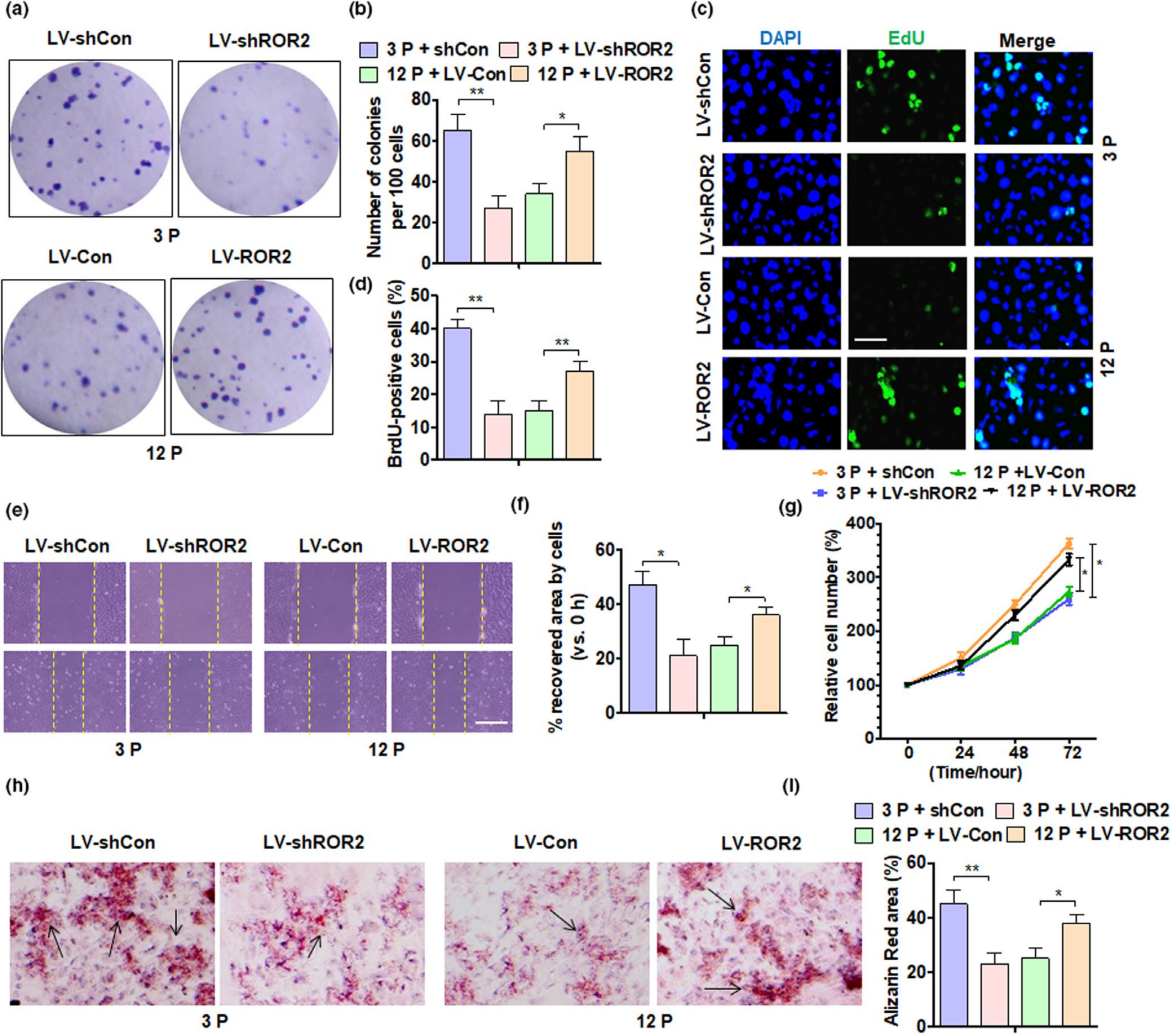
ROR2 depletion in young DPSCs inhibits while ROR2 overexpression in aged DPSCs restores their self‐renewal capability. (a) Colony‐forming unit (CFU) assay was performed in DPSCs from young donors at 3P and 12P after the indicated transfection, and crystal violet staining of the colonies was performed on day 14; all DPSCs were from young donors. (b) Quantitative analysis of CFU efficiency. (c) DPSCs (3P and 12P) treated as above and cell proliferation was detected by EdU staining; scale bar: 50 µm. (d) Quantitative analysis of EdU‐positive cells. (e) Representative scratch assay of DPSC (3P and 12P) treatment as above; scale bar: 200 µm. (f) Quantification of scratch bridging time and cell velocity. (g) DPSCs (3P and 12P) were transfected as above, and an MTS assay was performed to detect cell proliferation. (h and i) Alizarin Red staining and quantitative measurements of osteogenesis of DPSCs (3P and 12P) after indicated transfection. All data are expressed as Mean ± SEM from three independent experiments. For all analyses, *p < 0.05, **p < 0.01, and ***p < 0.001 compared to their corresponding controls

Since knocking down ROR2 can promote the senescence of DPSCs, we wanted to know whether ROR2 affects the self‐renewal capacity of DPSCs. First, we knocked down the expression of ROR2 in (3P) young DPSCs and overexpressed ROR2 in (12P) aged DPSCs. As shown in Figure S4a, depletion of ROR2 in young DPSCs decreased the expression of CD44, CD73, and CD105, but increased CD45 expression. However, overexpression of ROR2 in aged DPSCs elevated CD44 and CD105 but depressed CD45 expression. Moreover, ROR2 knockdown in young DPSCs inhibited, while its overexpression in aged DPSCs promoted clonal formation (Figure3a,b). We then used the BrdU assay to determine their cell proliferation ability and found that ROR2 knockdown in young DPSCs inhibited, while overexpression in aged DPSCs increased cell proliferation (Figure 3c,d). Additionally, cell scratch and MTS assay provided similar results (Figure 3e‐g). Moreover, deletion of ROR2 in (3P) DPSCs significantly declined mineralization and expression of osteogenesis‐related genes, whereas ROR2 overexpression increased the mineralization potential and osteogenesis‐related gene expression (Figure 3h,i, Figure S4b). Similar results were found for adipogenesis‐related gene expression in ROR2‐depleted or ROR2‐overexpressed DPSCs (Figure S4c). Collectively, these results indicated the role of ROR2 in regulating the self‐renewal capacity of DPSCs.

### 2.4 Sphingomyelin is involved in the senescence of DPSCs regulated by ROR2

**FIGURE 4:**
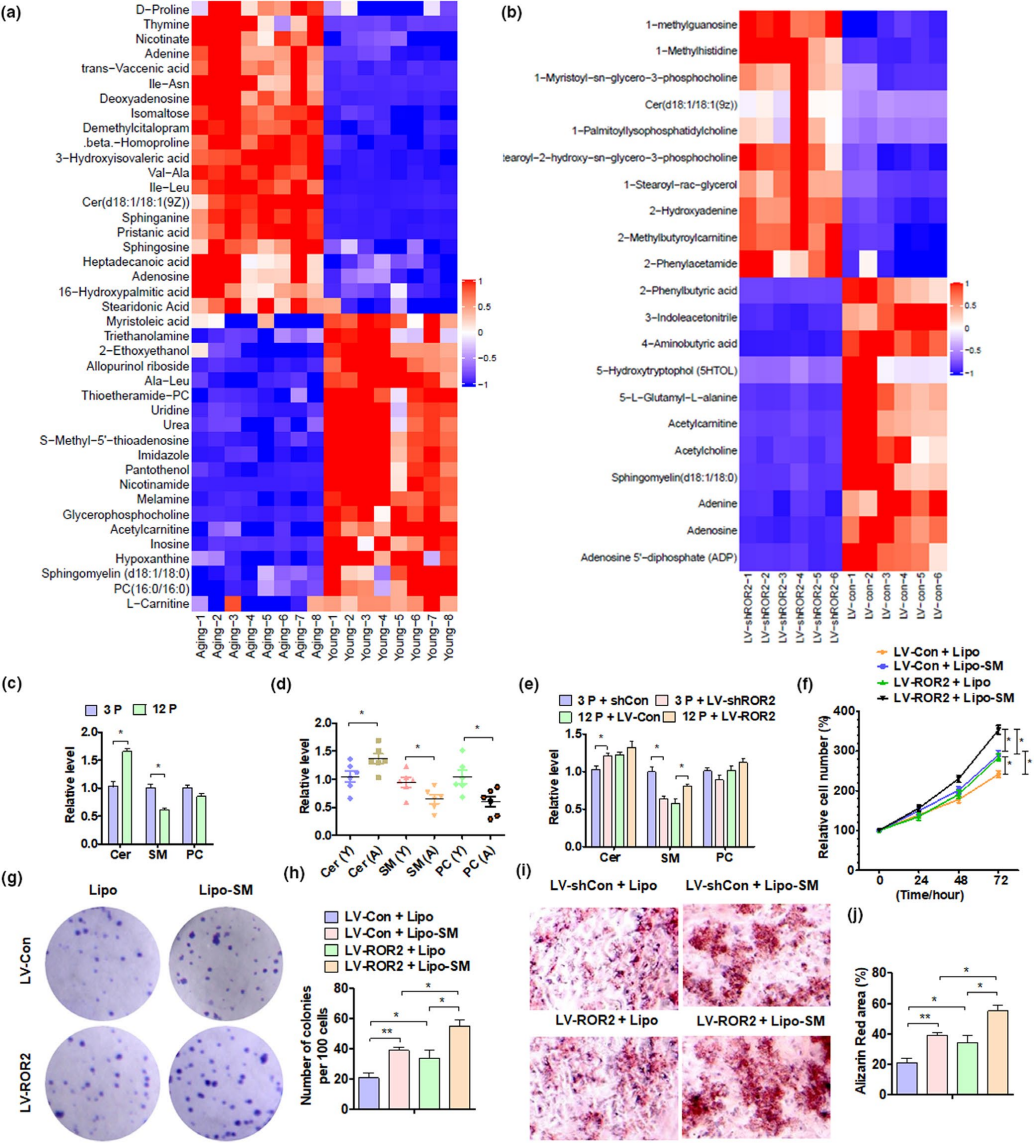
SM is involved in cell aging regulated by ROR2. (a and b) Heatmap of the metabolite signal intensity averaged across pulp tissues of 16 individuals (eight young and eight aged) and DPSCs from young donors (3P); depletion of ROR2, showing the top (a) 42 and (b) 21 altered metabolites. (c) MALDI‐MS/MS identified Cer, SM, and phosphatidylcholine (PC) levels in DPSCs at 3P and 12P. (d) The levels of Cer, SM, and PC were measured in young (Y, n = 6) and aged (A, n = 6) pulp tissues. (e) The levels of Cer, SM, and PC were detected in DPSCs from young donors (3P and 12P) after transfection. (f) DPSCs from young donors (12P) were transfected with ROR2 overexpression vector or lipo‐SM (lipofectamine‐SM), and an MTS assay was performed to detect cell proliferation. (g and h) CFU assay was performed with DPSCs from young donors (12P) after transfection, and crystal violet staining of the colonies was performed on day 14. (i and j) Alizarin Red staining and quantitative measurements of the osteogenesis of DPSCs from young donors (12P) after transfection. All data are expressed as Mean ± SEM from three independent experiments. For all analyses, *p < 0.05, **p < 0.01, and ***p < 0.001 compared to their corresponding controls

A large number of studies have shown that the process of cell metabolism is closely related to the aging of stem cells (Herranz & Gil, 2018; Ren et al., 2017). To investigate the metabolism of aged DPSCs, we analyzed the metabolism of pulp tissue from eight elderly and eight young donors by metabolomics analysis. Additionally, to understand how ROR2 regulates the senescence of DPSCs by regulating cellular metabolism, we also performed a cell metabolomics analysis in ROR2‐depleted DPSCs. From these two sets of metabolomics analyses, we found that both ceramide (Cer) and SM, which are a part of the lipid metabolism pathway, have significant differences between young and aging cells (Figure 4a,b). Using mass spectrometry, we confirmed that Cer was upregulated while SM was downregulated in aged DPSCs (Figure 4c). Similar results were found in human pulp tissues (Figure 4d). Furthermore, we knocked down ROR2 in 3P cells and overexpressed it in 12P cells, and detected SM and Cer expression. The result showed that ROR2, but not Wnt5a, knockdown in 3P cells inhibited SM and promoted Cer expression, while ROR2 overexpression in 12P cells only increased the amount of SM, but did not affect the amount of Cer (Figure 4e, Figure S5a). Studies have reported that SM promotes cell proliferation and maintains cell stemness, and its downregulation is important for cell aging (Ishida et al., 2016; Noh et al., 2019). We then overexpressed ROR2 in aged cells and treated them with SM simultaneously. An MTS assay showed that SM treatment significantly increased cell proliferation, and this process was further enhanced when transfected with ROR2 in DPSCs (Figure 4f). Colony formation analysis further confirmed these results (Figure 4g,h). Additionally, Alizarin Red staining showed that both SM treatment and ROR2 overexpression enhanced the osteogenic differentiation ability of DPSCs, while simultaneous treatment further enhanced the process (Figure 4i,j). The above results indicate that SM is involved in DPSC senescence regulated by ROR2, and SM supplementation can maintain the proliferation of DPSCs.

### 2.5 SMS1 mediates ROR2 to regulate the biogenesis of SM in DPSCs

**FIGURE 5:**
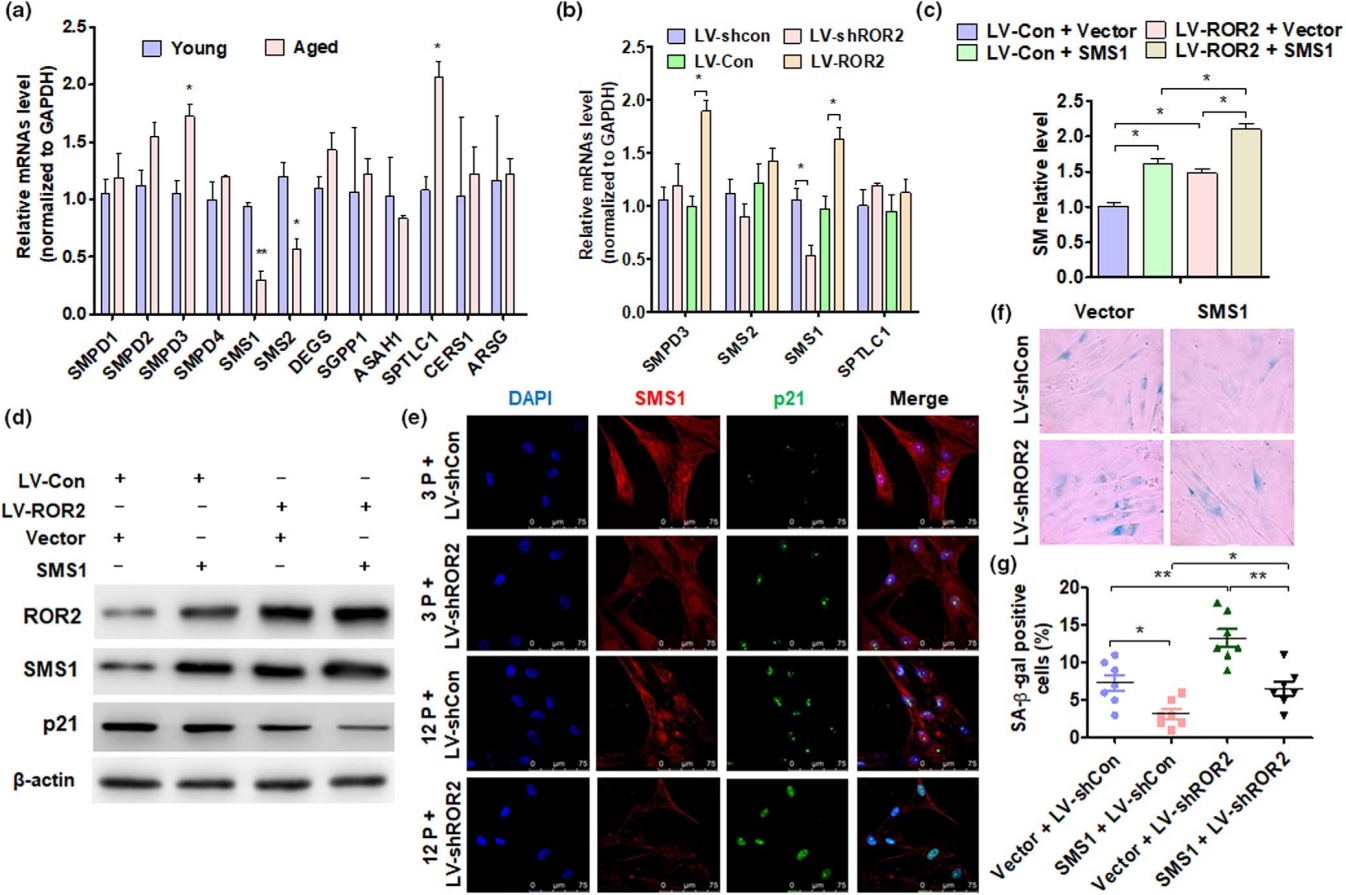
SMS1 mediates ROR2 to regulate SM production. (a) RT‐qPCR was performed to detect the candidate gene expression in young and aged pulp tissues. (b) RT‐qPCR was performed to detect the SMPD3, SMS1, SGMS2, and SPTLC1 gene expression in DPSCs at 12P after being transfected with the indicated vectors. (c) DPSCs at 12P were transfected with ROR2 or SMS1 overexpression vector or control vector, and SM level was measured with a capillary column. (d) The protein levels of ROR2, SMS1, and p21 were detected by Western blot analysis in LV‐ROR2, pcDNA3.1‐SMS1 (SMS1), or control vector‐transfected DPSCs (12P). (e) Immunofluorescence staining for SMS1 and p21 in DPSCs at 3P and 12P after being transfected with LV‐shCon or LV‐shROR2; scale bar: 100 µm. (f and g) DPSCs at 12P were transfected with LV‐shROR2, pcDNA3.1‐SMS1 vector, or their control vectors, and SA β‐gal staining was performed. For all analyses, *p < 0.05, **p < 0.01, and ***p < 0.001 compared to their corresponding controls

Studies have confirmed that both Cer and SM belong to the sphingolipid signaling pathway (Bienias et al., 2016). The biosynthesis and metabolism of SM involve multiple enzymes and reactions (Brodowicz et al., 2018). Our previous findings indicated that the expression of SM is downregulated in aged DPSCs. To determine, which genes are related to the sphingolipid signaling pathway and SM metabolism, we first detected 12 candidate genes related to SM metabolism in dental pulp tissues. The RT‐qPCR results showed that the expression of SMS1 and SMS2 was downregulated in aged pulp tissues, while the expression of SPTLC1 and SMPD3 was upregulated (Figure 5a). To verify whether the expression of these genes is related to ROR2, we knocked down ROR2 in 3P cells and overexpressed ROR2 in 12P cells and measured the expression of the above genes. The results showed that only SMS1 was downregulated in ROR2‐depleted DPSCs and upregulated in ROR2‐overexpressed DPSCs (Figure 5b). These results indicated that ROR2, but not Wnt5a, regulates the expression of SMS1, the key enzyme that promotes SM biogenesis in cells (Figure S5b). To determine whether SMS1 mediates ROR2 to regulate SM synthesis, we overexpressed ROR2 and SMS1 simultaneously. The results showed that overexpression of SMS1 or ROR2 independently promoted the synthesis of SM, which was enhanced with the simultaneous overexpression of SMS1 and ROR2 (Figure 5c). Moreover, SMS1 overexpression significantly inhibited the expression of p21, and this inhibition was further enhanced by the simultaneous overexpression of SMS1 and ROR2 in DPSCs (Figure 5d). Immunofluorescence staining also showed that SMS1 expression was downregulated in ROR2‐depleted 3P cells, and SMS1 expression was further reduced by ROR2 knockdown in 12P cells (Figure 5e). Previous studies have shown that SMS1 is related to cell senescence (Sogaard et al., 2019; Stoffel et al., 2016). We found that both SMS1 and ROR2 regulated cell senescence and proliferation of DPSCs. A β‐gal assay showed that SMS1 overexpression significantly reversed cell senescence and proliferation induced by ROR2 knockdown (Figures 5f,g, Figure S6). The above results suggest that the downregulation of ROR2 inhibits the expression of SMS1, thereby reducing the production of SM during the senescence of DPSCs.

### 2.6 The STK4‐FOXO1 axis mediates ROR2 to regulate SMS1 expression in DPSCs

**FIGURE 6:**
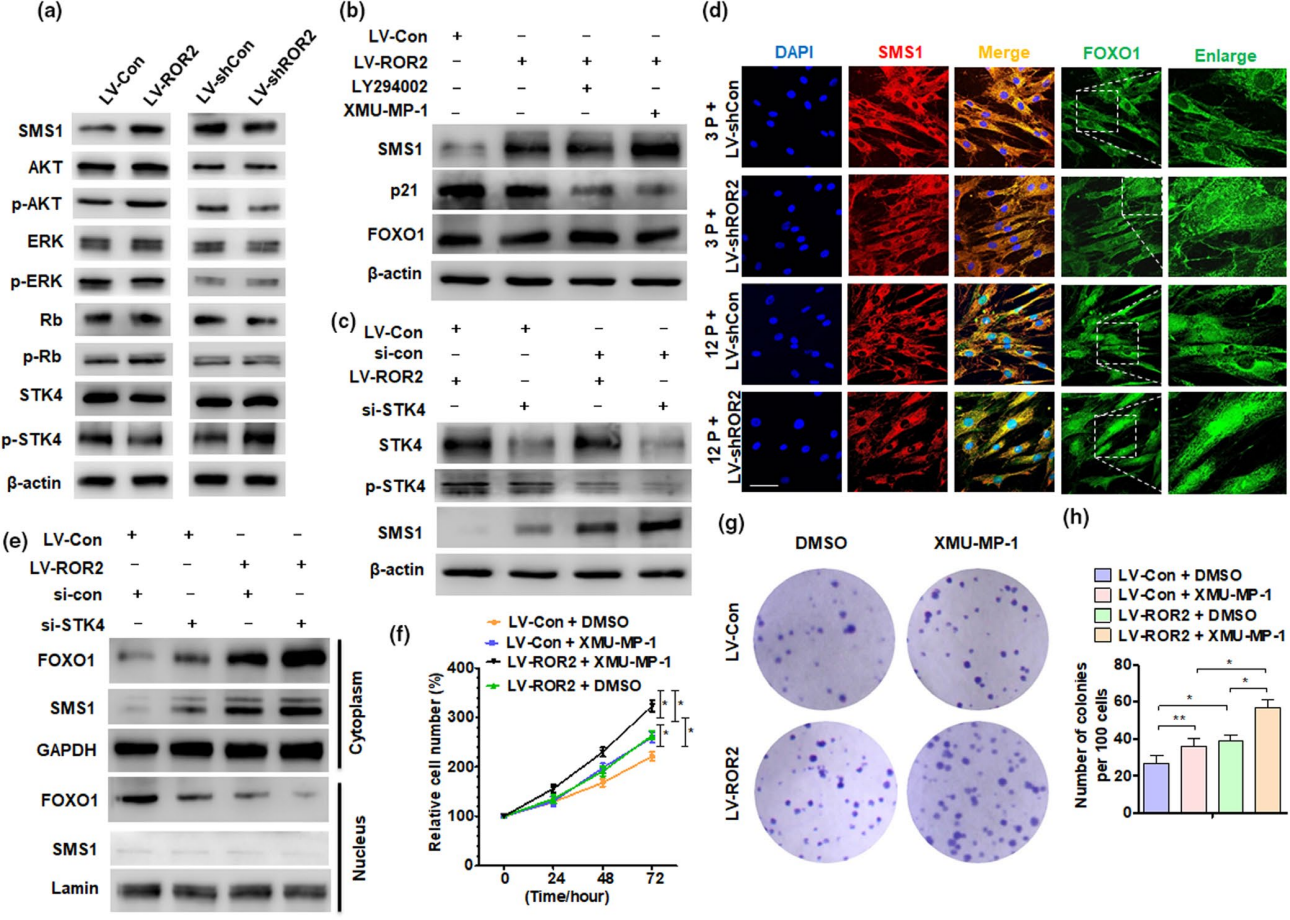
STK4‐FOXO1 axis mediates ROR2‐regulated SMS1 expression in DPSCs. (a) DPSCs at 3P were transfected with LV‐shCon and LV‐shROR2, while at 12P were transfected with LV‐Con and LV‐ROR2; Western blot analysis was performed to detect the indicated protein expression. (b) DPSCs (12P) were transfected with LV‐ROR2 or LV‐Con, and treated with AKT pathway inhibitor (LY294002) or STK4 inhibitor (XMU‐MP‐1) for 2 h; Western blot analysis was performed to detect SMS1, p21, and FOXO1 protein expression. (c) DPSCs (12P) were transfected with LV‐ROR2, si‐STK4, or their control vectors, and RNA; Western blot analysis was performed to detect the indicated protein level. (d) Immunofluorescence staining for SMS1 and FOXO1 in DPSCs at 3P and 12P after being transfected with LV‐shROR2 or their control vector; scale bar: 100 µm. (e) Western blot analysis was performed to detect FOXO1 and SMS1 in subcellular fractionations of DPSCs (12P) transfected with LV‐ROR2, si‐STK4, or their control vector, and RNA; Lamin and GAPDH served as controls for the nuclear and cytoplasmic fractions, respectively. (f) DPSCs at 12P were treated with XMU‐MP‐1 after LV‐ROR2 transfection for 48 h, and an MTS assay was performed to detect cell proliferation. (g and h) CFU assay was performed in DPSCs at 12P after transfection and treatment, and crystal violet was used to stain the colonies on day 14. DPSCs at passages 3–12 used in these experiments were from young donors. For all analyses, *p < 0.05, **p < 0.01, and ***p < 0.001 compared to their corresponding controls

To understand how ROR2 regulates the expression of SMS1, 3P DPSCs were transfected with LV‐shCon and LV‐shROR2, while 12P DPSCs were transfected with LV‐Con and LV‐ROR2. A Western blot analysis was performed to detect the signal pathway protein expression. As shown in Figure 6a, p‐AKT was significantly elevated while p‐STK4 was inhibited in DPSCs in which ROR2 was overexpressed; these results were reversed in DPSCs with depleted ROR2. The 12P DPSCs were then transfected with LV‐ROR2 or LV‐Con, and treated with an AKT pathway inhibitor (LY294002) or an STK4 pathway inhibitor (XMU‐MP‐1). A Western blot analysis showed that both LY294002 and XMU‐MP‐1 depressed p21 expression, and only XMU‐MP‐1 treatment increased the level of SMS1 protein after ROR2 overexpression. However, these did not affect the expression of FOXO1 (Figure 6b). Interestingly, treatment with the two PI3K inhibitors (MK2206 and LY294002) decreased the FOXO1 (s319) protein level but did not change the expression of FOXO1 (s212) or SMS1. However, the STK4 inhibitor XMU‐MP‐1 decreased FOXO1 (s212) and increased SMS1 protein level, but did not affect FOXO1 (s319) levels (Figure S7). We speculate that the degree of phosphorylation of FOXO1 (s212) may determine the expression of SMS1. Furthermore, we knocked down STK4 or overexpressed ROR2. A Western blot analysis showed that ROR2 overexpression did not affect the expression of STK4 protein level, but decreased p‐STK4 level. STK4 knockdown promoted SMS1 expression, and this effect was enhanced with the simultaneous knockdown of STK4 and overexpression of ROR2 in DPSCs (Figure 6c). Previous studies have shown that FOXO1 is involved in the expression of senescence genes regulated by ROR2 (Endo et al., 2020). Subsequently, immunofluorescence staining was done for SMS1 and FOXO1 in 3P and 12P DPSCs after transfection with LV‐shROR2. Interestingly, we found that ROR2 knockdown reduced the expression of SMS1, but increased the nuclear localization of FOXO1, especially in 12P DPSCs, which had more FOXO1 in the nucleus (Figure 6d, Figure S8). To verify this, Western blot analyses were performed to detect FOXO1 and SMS1 in subcellular fractionations of DPSCs. The results showed that STK4 knockdown or ROR2 overexpression independently increased the nuclear localization of FOXO1, and the effect was enhanced with the simultaneous knockdown of STK4 and overexpression of ROR2 (Figure 6e). These results suggested that the inhibition of the STK4 pathway may increase the expression of SMS1 by promoting FOXO1 nuclear localization. Next, DPSCs were treated with XMU‐MP‐1 after LV‐ROR2 transfection, and cell proliferation was detected by MTT assay and colony‐forming assay. As shown in Figure 6f–h, XMU‐MP‐1 treatment significantly enhanced cell growth, and ROR2 overexpression further promoted cell proliferation. Collectively, our findings suggested that ROR2 attenuates DPSC senescence, at least in part, by repressing STK4 signaling during tooth aging.

### 2.7 Forkhead Box O1 directly regulates the transcription of SMS1 in DPSCs

**FIGURE 7:**
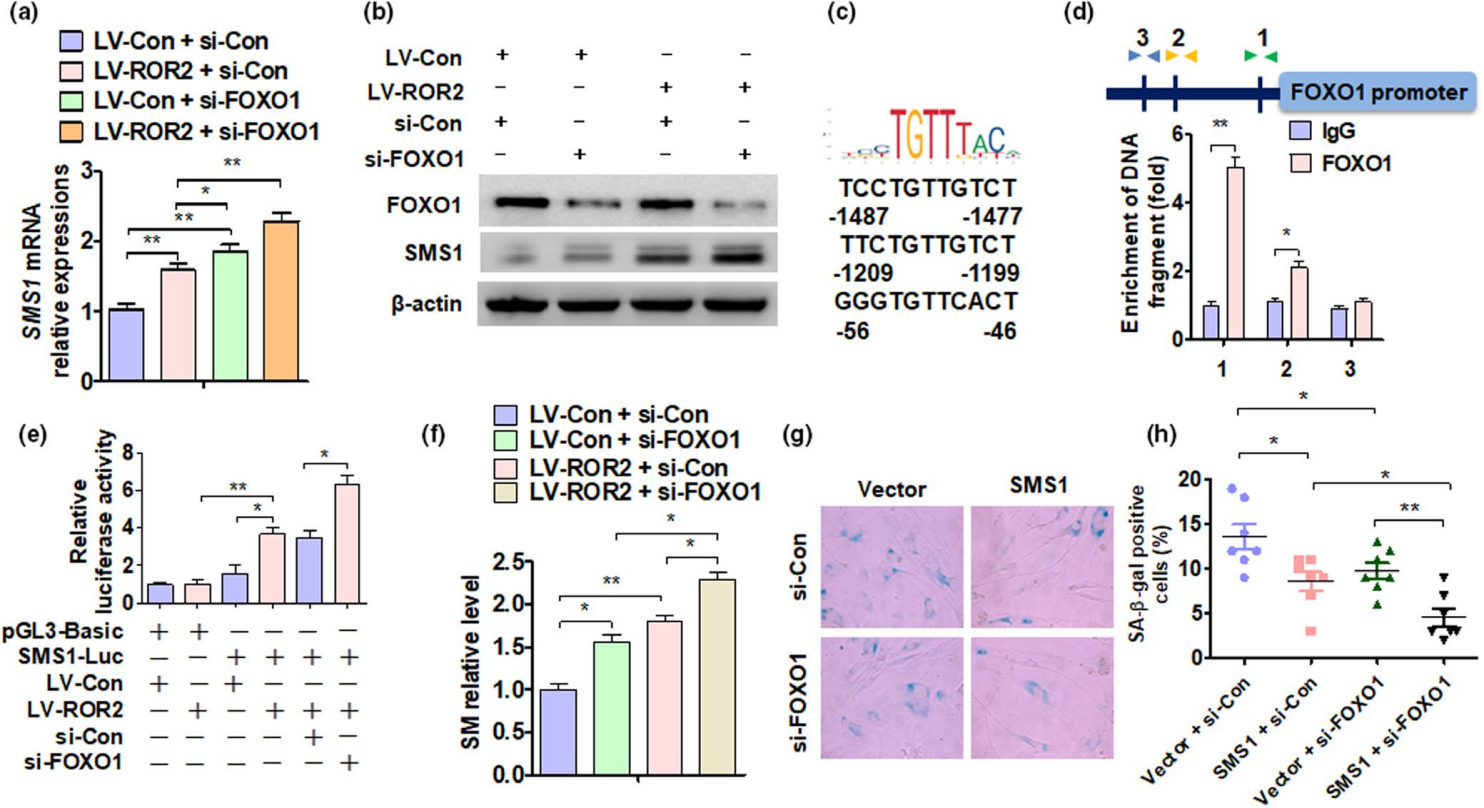
Forkhead Box O1 directly regulates SMS1 transcription. (a and b) DPSCs at 12P were transfected with LV‐ROR2, si‐FOXO1 vector, or their control vectors, and SMS1 level was measured by (A) RT‐qPCR and (B) Western blot analysis. (c) Three putative SMS1 binding elements within the 2‐kb promoter region of FOXO1 were identified. (d) ChIP‐qPCR was used to determine FOXO1‐SMS1 binding to the SMS1 promoter region in DPSCs (12P); **p < 0.01 compared to IgG. (e) T cells (n = 293) were transfected with the indicated constructs; luciferase activity was measured by dual‐luciferase reporter assays. (f) DPSCs at 12P were transfected with LV‐ROR2, si‐FOXO1 vector, or their control vectors, and the level of SM was measured. (g and h) DPSCs at 12P were transfected with si‐FOXO1, pcDNA3.1‐SMS1 vector, or their control vectors, and SA β‐gal staining was performed. DPSCs at passages 3–12 used in these experiments were from young donors. For all analyses, *p < 0.05, **p < 0.01, and ***p < 0.001 compared to their corresponding controls, unless specified

To determine whether FOXO1 directly regulates SMS1 transcription, we first detected the mRNA level of SMS1 in DPSCs after transfection with LV‐ROR2 and si‐FOXO1. The results showed that ROR2 overexpression and FOXO1 knockdown independently increased the mRNA level of SMS1; the effect was enhanced when both treatments were applied simultaneously (Figure 7a). A Western blot analysis showed the same result after DPSCs were transfected with LV‐ROR2 and si‐FOXO1 (Figure 7b). Then, the Ensembl and Promo 3.0 websites were used to determine the potential binding sites of SMS1 on the 2‐kb 5' promoter region. We found three potential binding elements within this region (Figure 7c). ChIP analysis confirmed that FOXO1 bound predominantly to the −46 to −56 and −1199 to −1209 nt of the SMS1 promoter (Figure 7d). The luciferase reporter further confirmed the result of the ChIP analysis (Figure 7e). These results showed the critical role of FOXO1 in the regulation of DPSC senescence by promoting SMS1 transcription. To determine whether FOXO1 mediates ROR2 to regulate SM expression, DPSCs were transfected with LV‐ROR2 or si‐FOXO1. The results showed that FOXO1 knockdown alone promoted the expression of SM, and with ROR2 overexpression, the expression of SM increased further (Figure 7f). To determine whether FOXO1 and SMS1 simultaneously regulated DPSC senescence, cells were transfected with si‐FOXO1 or pcDNA3.1‐SMS1 vector. An SA β‐gal analysis showed that the overexpression of SMS1 significantly inhibited cell senescence, while the simultaneous knockdown of FOXO1 further depressed cellular senescence (Figure7g,h). Thus, FOXO1 regulates SM expression and cellular senescence by directly promoting SMS1 transcription.

## 3 DISCUSSION

In this study, we investigated the biological role of the ROR2/STK4‐FOXO1/SMS1 axis in regulating SM biogenesis and senescence of DPSCs. First, ROR2 expression was found to be significantly decreased in aged pulp tissues and DPSCs isolated from elderly donors. Second, ROR2 was shown to restore the self‐renewal capabilities of aged DPSCs. Third, ROR2 positively regulated SM biosynthesis, which promoted DPSC proliferation and self‐renewal. Mechanistically, ROR2 reduced STK4 phosphorylation which promoted FOXO1 translocation to the nucleus. FOXO1 bound to the promoter of SMS1 directly, which catalyzed SM biogenesis and repressed its transcription.

ROR2 is an important gene for bone development. We have previously shown that a novel heterozygous compound variation in the ROR2 gene leads to ARRS, which expands the mutation spectrum of the disorder (Yang et al., 2020). This indicates that ROR2 plays a role in cell proliferation and differentiation, developmental morphogenesis, and tissue/organogenesis. Besides, ROR2 depletion in NIH/3T3 cells results in an increased expression of aging‐related genes, such as p21^cip1^ and p27^kip1^ (Endo et al., 2020). However, the role of ROR2 in DPSC senescence and self‐renewal is still unclear. Interestingly, our results indicated that the expression of ROR2 in DPSCs declines with age, and ROR2 overexpression attenuates cellular senescence in aged DPSCs. ROR2 regulates DPSC senescence and is partially dependent on the cell cycle inhibitor p21cip1; thus, p21cip1 could be an ideal marker for DPSC senescence. ROR2 is the receptor of Wnt5a and part of the non‐canonical WNT pathway (Hasegawa et al., 2018; Yu et al., 2016). In this study, we found that the expression of Wnt5a was significantly downregulated in aging DPSCs and tissues from the elderly donors. However, Wnt5a did not participate in ROR2‐regulated SMS1 expression or SM biogenesis in DPSCs.

Stem cell senescence is a complex phenotype, which includes changes in the ability of cells to function and replicate, and is characterized by morphological expansion, flattening, and irreversible G1 growth arrest (Ma et al., 2019). It has been reported that senescence may lead to the stagnation of tissue regeneration, eventually resulting in organ failure and death (Carlos Sepulveda et al., 2014). Self‐renewal, migration, and osteogenic differentiation are reduced in aged DPSCs, and these changes reduce dental healing and regeneration (Yi et al., 2017). A recent study used the passage of stem cells to obtain senescent DPSCs, and the six passage‐DPSCs had significantly high levels of senescence indicators compare to three passage‐DPSCs (Ma et al., 2019). In our study, we used DPSCs at the third, sixth, and twelfth passages to determine their aging mechanism. A study reported that DPSCs with low proliferative capability also showed positive expression of the nerve growth factor receptor p75 (CD271) (Alraies et al., 2017). Our results demonstrated that the expression of ROR2 in aged DPSCs was downregulated. The downregulation of ROR2 suppressed the expression of SM through the STK4‐FOXO1/SMS1 axis. The downregulation of SM expression is related to the senescence of a variety of cells, including DPSCs (Ishida et al., 2016; Melum et al., 2019).

Forkhead Box O1 belongs to the forkhead family of transcription factors, which are characterized by a distinct forkhead domain; the specific function of this gene has not yet been determined. However, it may play a role in myogenic growth and differentiation (Kodner et al., 2008). FOXO1 has numerous targets, such as genes involved in apoptosis and autophagy, cell cycle arrest, anti‐oxidative enzymes, and metabolic and immune regulators (Murtaza et al., 2017; Wang et al., 2016). STK4 (MST1) phosphorylates FOXO1 proteins at a conserved site within the forkhead domain and promotes FOXO1 nuclear translocation, thereby inducing cell death‐related gene expression (Lehtinen et al., 2006). Some studies have shown that both the PI3K/AKT signaling pathway and STK4 pathway regulate the nuclear localization of FOXO1 through phosphorylation of FOXO1. However, the two pathways have different phosphorylation sites for FOXO1. PI3K/AKT generally phosphorylates the s319 site of FOXO1, while STK4 acts on the s212 site (Chen et al., 2018; Choi et al., 2009; Kim et al., 2019; Pan et al., 2017). In this study, we found that ROR2 regulated the STK4‐FOXO1 cascade and increased FOXO1 nuclear translocation. Then, FOXO1 bound directly to the SMS1 promoter and regulated its expression. Furthermore, SMS1 upregulation promoted SM biogenesis and slowed down the senescence of DPSCs. We also found that treatment with two PI3K inhibitors (MK2206 and LY29400) decreased the FOXO1 (s319) protein level, but did not change the expression of FOXO1 (s212) and SMS1. However, the STK4 inhibitor XMU‐MP‐1 decreased both SMS1 and FOXO1 (s212) protein levels, but not FOXO1 (s319) levels. We speculate that the phosphorylation level of FOXO1 (s212) may determine the expression of SMS1. Next, we aim to explore the role of epigenetic modifications at different sites of FOXO1 in the senescence of DPSCs.

Studies have reported that stress‐induced senescence refers to the process of cellular senescence in which freshly isolated cells adapt to the artificial in vitro environment during their growth (Morsczeck, 2019). Stress factors lead to shocks in the cell culture and ultimately cause premature aging (Sherr & DePinho, 2000). These include changes in nutrient and growth factor levels, loss of extracellular matrix components and surrounding cell types, and increased oxygen levels (Kuilman et al., 2010). These stress factors can activate the tumor suppressor p53 and important cell cycle inhibitor kinases to accelerate the process of cellular senescence through the MAP/p38 pathway (Son et al., 2011). In this study, we found that p53 expression is upregulated in senescent DPSCs; knocking down ROR2 promotes p53 expression. To determine whether p53 participates in SM biogenesis and DPSC senescence by regulating the ROR2/STK4/FOXO1/SMS1 axis, we overexpressed or knocked down p53 in DPSCs. The results showed that the overexpression of p53 was suppressed, and the knockdown of p53 promoted the expression of ROR2 (Figure S9). Therefore, we speculate that stress‐induced p53 expression may be important for the regulation of the ROR2/STK4/FOXO1/SMS1 axis during SM biogenesis and DPSC senescence.

In conclusion, our study demonstrated that ROR2 expression in DPSCs declined with age. ROR2 overexpression restored the differentiation and regeneration of DPSCs. Moreover, the ROR2/STK4‐FOXO1/SMS1 axis regulated SM biogenesis and DPSC senescence. Our results collectively indicated that ROR2 could be an ideal target for antagonizing aging in DPSCs.

## 4 EXPERIMENTAL PROCEDURES

### 4.1 Cell isolation, culture, and transfection

The study was approved by the Ethics Committee of the Beijing Stomatology Hospital, Capital Medical University. Human dental pulp tissues were collected from clinically healthy teeth extracted for impaction or other orthodontic reasons (young donors), while those extracted for trauma or other reasons (old donors) were obtained after informed consent. Human DPSCs were classified into two groups: young (15–22 years old) and elderly (56–72 years old). DPSCs were cultivated and verified according to our previous protocol (Gronthos et al., 2000). The surface of the tooth was cleaned, the pulp cavity was exposed by using a sterile dental crack drill, and an incision was made at the cementum‐enamel junction. The pulp tissue was gently separated from the crown and root and was digested in a solution of 3 mg/ml Type I collagenase (Sigma‐Aldrich; Merck KGaA) and 4 mg/ml dispase (Sigma‐Aldrich) at 37°C for 1 h. A single‐cell suspension was obtained by passing the cells through a 70‐µm filter (Falcon). The culture media were changed every 3 days. The DPSCs at passages 3–12 were used in subsequent experiments (Liang et al., 2020; Yi et al., 2017). Unless mentioned otherwise, all the DPSCs at passages 3–12 were from young donors. For SM transfection, 125 µM sphingomyelin (Sigma, #85615) and 10 µl Lipofectamine (Thermo, STEM00003) were mixed thoroughly. The mixture was then incubated at room temperature for 15–20 min to form Lipo‐SM. After a change of the medium, the cells were incubated at 37°C for 72 h in the presence of Lipo or Lipo‐SM (Ishida et al., 2016).

### 4.2 Western blot analysis

The protein was extracted from the cultured DPSCs with RIPA lysis buffer as previously described (Yang et al., 2019; Yang et al., 2017). Then, 8% or 10% SDS‐PAGE was used to separate the proteins and electro‐transferred to a PVDF membrane (Millipore). After blocking using 5% milk in TBS for 2 h, primary antibodies were used to incubate the membranes at 4°C overnight. Then, the blots were detected by ECL (enhanced chemiluminescence) FuazonFx (VilberLourmat). The antibodies used were anti‐FOXO1 (1:1000), anti‐SMS1 (1:1000), anti‐p16 (1:1000), anti‐p21 (1:1000), anti‐p53 (1:1000), anti‐ROR2 (1:1000), anti‐STK4 (1:1000), anti‐p‐STK4 (1:1000), anti‐AKT (1:1000), anti‐p‐AKT (1:1000), anti‐ERK (1:1000), anti‐P‐ERK (1:1000), anti‐RB (1:1000), anti‐p‐RB (1:1000), and anti‐β‐actin (1:1000). Images were captured and processed by FusionCapt Advance Fx5 software (VilberLourmat). All experiments were replicated three times independently.

### 4.3 RNA extraction and RT‐qPCR

The DPSCs were lysed using QIAzolLysis Reagent (QIAGEN). The concentration and purity of the RNA were determined using NanoDrop 2000 (Thermo Fisher) (Sun et al., 2017). For mRNA analysis, reverse RNA transcription was performed using the M‐MLV First‐Strand Kit (Life Technologies). The Platinum SYBR Green qPCR SuperMix‐UDG Kit (Invitrogen) was used for qRT‐PCR of mRNAs. The RT‐PCR experiments were done on a CFX96^™^ Real‐Time System (Bio‐Rad). All the primers used in this study have been listed in Table S1. The expression levels were normalized to the GAPDH gene using the 2^–△△Ct^ method.

### 4.4 MTS assay

A total of 1 × 10^4^ cells were seeded into 96‐well plates. After 24 h, the cells received corresponding treatments according to the experimental design. Then, the medium was removed, and the cells were washed with phosphate‐buffered saline (PBS). Thereafter, 2 mg/ml MTS reagent was added to Hank's buffer and incubated for 1 h until dark blue crystals were observed in the cytoplasm under an optical microscope. The crystal was dissolved in DMSO, after, which, the absorbance was measured using a 490‐nm thermal fluorescence scanning Ascent spectrometer, with background subtraction at 650 nm.

### 4.5 Colony formation assay

A total of 100 DPSCs were seeded into 6‐well plates, cultured for 1 week, and fixed with glacial acetic acid/methanol solution. Thereafter, the colony was stained with 0.5% crystal violet, and the colonies formed were analyzed under a microscope.

### 4.6 SA β‐gal staining and quantitative analysis

Cells were seeded into 12‐well plates incubated at 37°C in 5% CO_2_ for 12 h. Then, the cells were fixed for 15 min, washed with PBS, and incubated with a staining mixture at 37°C for 18 h. The staining mixture was from the SA‐β‐gal staining kit (Abcam). Then, SA β‐gal staining quantification was done using the Image‐Pro Plus 6.0 program (Media Cybernetics).

### 4.7 Metabolite profiling

Human dental pulp tissue from healthy teeth of young (n = 8) and elderly (n = 8) donors were dissolved in PBS and stored at −80°C. pLV‐hU6‐ROR2 shRNA3 (Human)‐hef1a‐mNeongreen‐P2A‐Puro (LV‐ROR2) was purchased from Syngentech Co., LTD. DPSCs at passage 3 (5 × 10^5^) were cultured in flasks. After 12 h, the DPSCs were infected with lentiviral particles containing LV‐shROR2 (30 μl, 1.5 × 10^9^ TU/ml) or LV‐shcon. The following day, the DPSCs were cultured in the medium containing puromycin (1 μg/ml). About 1 × 10^7^ cells from each group were washed thrice with PBS. Then, every sample was mixed with 1 ml of cold methanol/acetonitrile/H_2_O (2:2:1, v/v/v) and adequately vortexed. The homogenate was sonicated at 0°C (30 min/once, twice), incubated for 60 min at −20°C to precipitate the protein, and centrifuged (16,200 *g*, 4°C, 15 min). The supernatant was collected and dried under vacuum and stored at −80°C. The sample was redissolved in 100 µl of acetonitrile/water (1:1, v/v), adequately vortexed, and then centrifuged (18,800 *g*, 4°C, 15 min). The supernatant was collected for LC‐MS/MS analysis (Guo et al., 2020).

### 4.8 Osteogenic induction

To evaluate the osteogenic differentiation potential in vitro, 1 × 10^5^ DPSCs were seeded into 6‐well plates. The DPSCs received corresponding treatments according to the experimental design. The LvSubconfluent cultures were incubated in the osteogenic medium (Invitrogen) for 2 weeks. The medium was changed every 2 days. Then, the cells were fixed with 70% ethanol, and the potential for osteogenic differentiation of MSCs was determined by Alizarin Red staining. The mineralization was measured using the Image‐Pro Plus 6.0 program (Media Cybernetics).

### 4.9 Flow cytometry

The mesenchymal stem cell markers expressed by DPSCs were detected by flow cytometry (Jin et al., 2019). In short, the 6P (passage 6) DPSC was separated using 0.25% trypsin/EDTA (Gibco) and resuspended in PBS at a density of 1 × 10^6^ cells/ml. The selected cells were then incubated with the following fluorescent‐conjugated antibodies: CD105‐PE, CD73‐PE, CD34‐FITC, CD44‐FITC, CD45‐FITC, and isotype‐matched antibodies (all under shading) for 30 min. Unlabeled cells served as a negative control. The cells were then washed three times with PBS to remove unbound antibodies, and resuspended in 300 µl of PBS. The cells were analyzed using a flow cytometer (FACSCalibur; BD Biosciences), and the FlowJo software (FlowJo) was used for data analysis.

### 4.10 Quantification of SM

The amount of SM was measured as described in previous studies (Ishida et al., 2016; Rodriguez‐Alcala & Fontecha, 2010). Briefly, the sphingolipid extracts were dissolved in methanol, mixed, and sonicated at room temperature for 10 min. An HPLC analysis was performed with a phase column containing highly pure silica gel (Inertsil SIL‐100A; GL Science). The isocratic mobile phase was composed of acetonitrile–methanol–distilled water (65:21:14) with 0.1% (v/v) phosphoric acid, and the flow rate (1.3 ml/min) was accurately regulated. The UV detector was set at 210 nm.

### 4.11 Immunofluorescence staining

The cells were fixed with 4% formaldehyde for 15 min and washed with PBS. Then, the cell smears were preincubated with 10% normal goat serum (710027, KPL) for 30 min. The cell smears were incubated with the primary antibodies anti‐ROR2, anti‐SMS1, anti‐FOXO1, and anti‐P21 at 37°C for 2 h. The fluorescence‐labeled rabbit IgG antibody (021516, KPL) was used as the secondary antibody. Finally, the cell smears were incubated in DAPI for 15 min for nuclear counterstaining. Images were acquired using confocal microscopy (DM6000CFS, Leica) and digitized with LAS AF software.

### 4.12 Statistical analysis

All data are reported as mean ± SD. Each experiment was repeated at least three times independently. The differences in mean values between the two groups were assessed by the Student's t test. Statistically significant differences were evaluated at *p* < 0.05.

## CONFLICT OF INTEREST

The authors declare no conflict of interest.

## AUTHOR CONTRIBUTIONS

C. G., X. C., Z. Y., and D. Z. contributed to investigation, data acquisition and curation, formal analysis, and visualization. X. D., X. C., and X. H. contributed to experimental performance and acquisition of data. K. Y. contributed to experimental design and visualization. J. W and S. W. contributed to resources. X. D. and Y. H. contributed to editing of manuscript and conception. X. C. contributed to formal analysis. D. Z., Z. Y., and K. Y. contributed to conception, experimental design, supervision, visualization, writing and editing of original manuscript, funding acquisition, and project administration.

## Supporting information

Fig S1‐S9

Table S1

## Data Availability

The data that support the findings of this study are available from the corresponding author upon reasonable request.
