# Supplementary material for: Downregulation of ROR2 promotes dental pulp stem cell senescence by inhibiting STK4‐FOXO1/SMS1 axis in sphingomyelin biosynthesis
Source: Aging Cell. 2021 Jul 18;20(8):e13430. doi: 10.1111/acel.13430 (PMC8373368; doi:10.1111/acel.13430)
Supplement: Supplementary file 1 — Fig S1‐S9 [file ACEL-20-e13430-s002.docx]

**Downregulation of ROR2 promotes dental pulp stem cell senescence by inhibiting STK4-FOXO1/SMS1 axis in sphingomyelin biosynthesis**

Xing-yue Dong^1#^, Yan-xia Huang^1#^, Xiao-yang Chu^2^, Jue Wu^3^, Shan Wang^3^, Xin He^1^, Chun-Yan Gao^1^, Xu Chen^1^, Kai Yang^4^*, Dong-liang Zhang^1^*

^1^ Department of Orthodontics, Beijing Stomatological Hospital, School of Stomatology, Capital Medical University, Beijing, China; ^2^ Department of Stomatology, Fifth Medical Center of Chinese PLA General Hospital, Beijing, China; ^3^ Translational Medical Research Center, Medical Innovation Research Division of Chinese PLA General Hospital, Beijing, China; ^4^ Prenatal Diagnosis Center, Beijing Obstetrics and Gynecology Hospital, Capital Medical University, Beijing, China.

**Supplementary Materials**


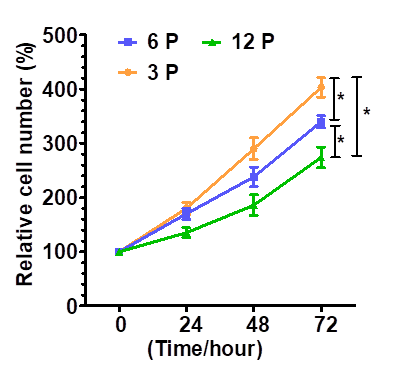


**Supplementary Figure S1.** MTS assay was performed to detect the proliferation of DPSCs at 3P, 6P, and 12P from young donors; *P < 0.05 compared to the corresponding control.

**Supplementary Table S1**


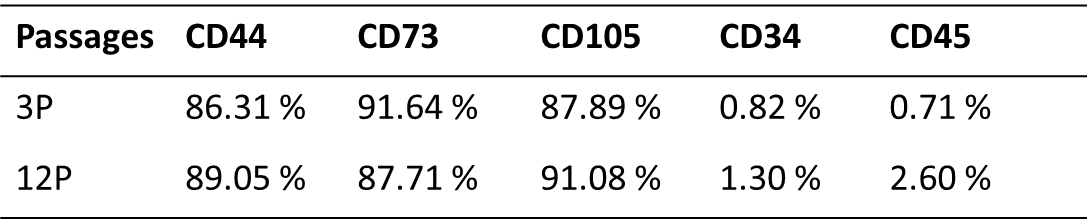


**Flow cytometry was performed to detect the expression of mesenchymal stem cell markers.** DPSCs at 3P or 12P were incubated with the fluorescent-conjugated antibodies (CD105-PE, CD73-PE, CD34-FITC, CD44-FITC, and CD45-FITC) and isotype-matched antibodies (all under shading) for 30 min. The cells were detected by a flow cytometer, and the data were analyzed using the FlowJo software.


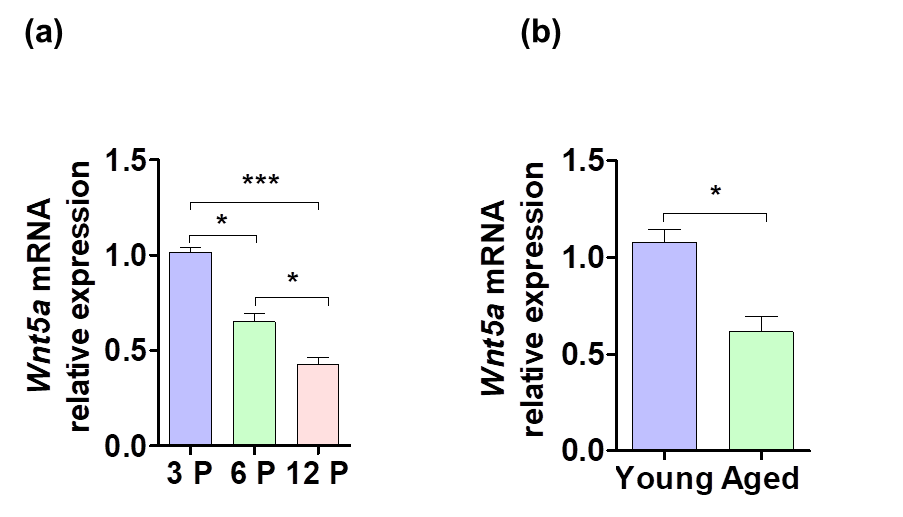


**Supplementary Figure S2. (a)** RT-qPCR was performed to detect Wnt5a mRNA expression in DPSCs at 3P, 6P, and 12P from young donors. **(b)** Wnt5a mRNA expression was measured in DPSCs at 6P from young or aged donors by RT-qPCR. For all analyses, *P < 0.05 and ***P < 0.001 compared to the corresponding control.


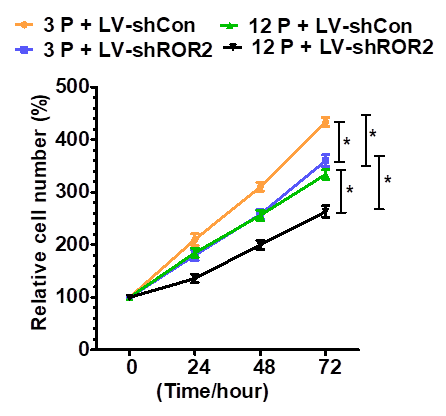


**Supplementary Figure S3.** DPSCs (3P and 12P) from young donors were transfected as indicated, and an MTS assay was performed to detect cell proliferation; *P < 0.05 compared to the corresponding control.


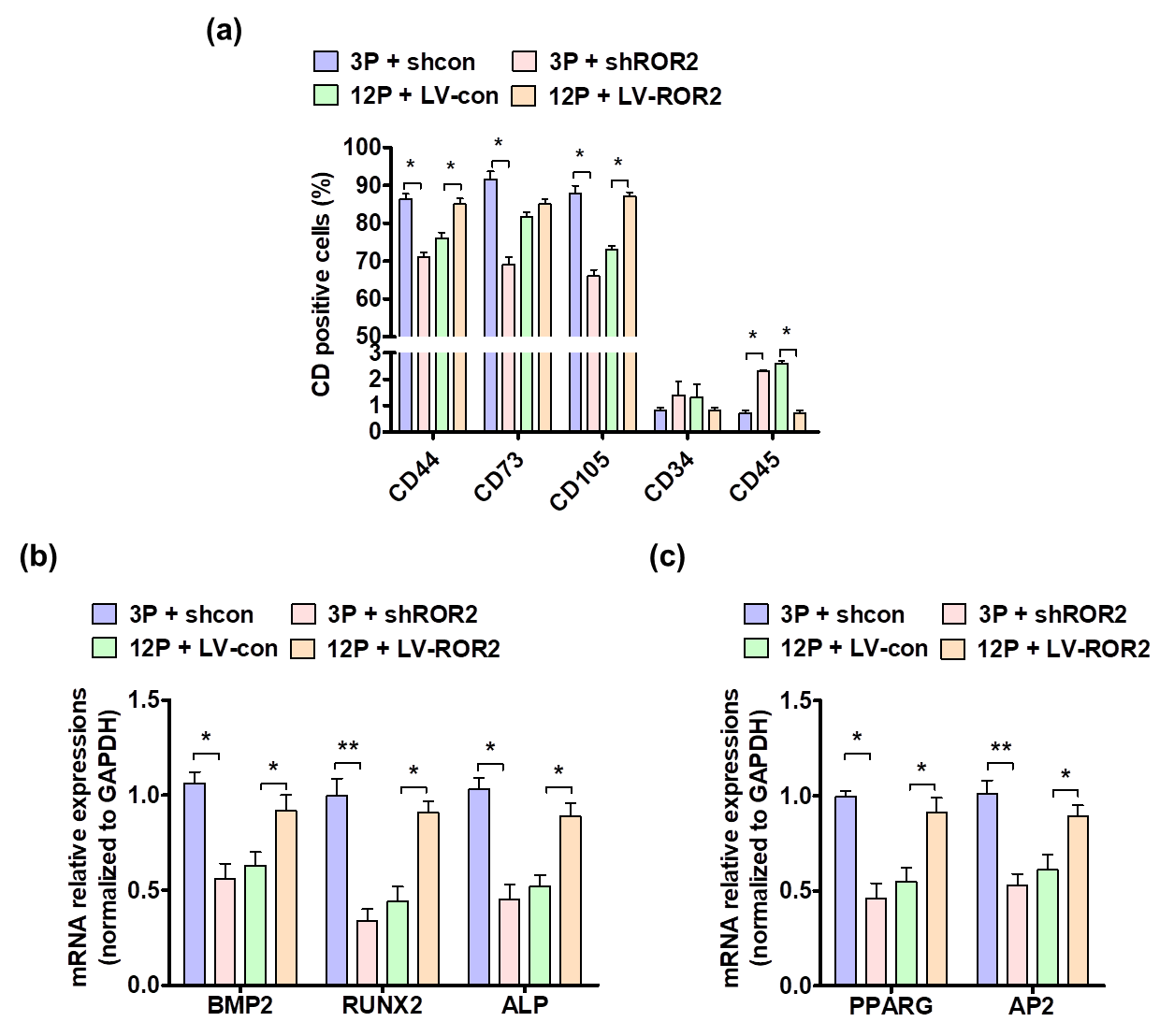


**Supplementary Figure S4. (a)** DPSCs (3P and 12P) from young donors were transfected as indicated, and flow cytometry was performed to examine CD44, CD73, CD105, CD34, and CD45 positive cells. **(b)** DPSCs (3P and 12P) from young donors were transfected as indicated, and RT-qPCR was performed to detect the BMP2, RUNX2, and ALP (osteogenesis-related genes) mRNA expression. **(c)** DPSCs (3P and 12P) from young donors were transfected as indicated, and RT-qPCR was performed to detect the PPARG and AP2 (angiogenesis-related genes) mRNA expression. For all analyses, *P < 0.05 compared to the corresponding control.


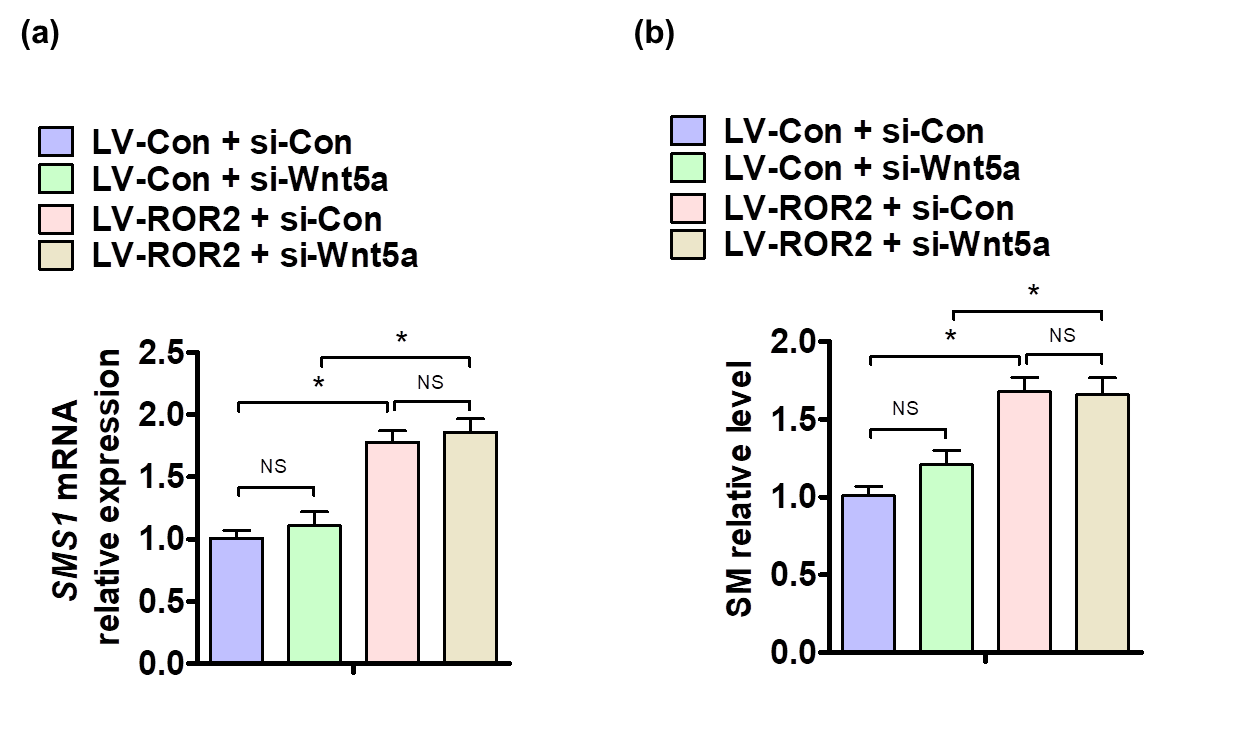


**Supplementary Figure S5. (a)** DPSCs (12P) from young donors were transfected as indicated; RT-qPCR was performed to detect SMS1 mRNA expression. **(b)** The levels of Cer, SM, and PC were detected in DPSCs from young donors (12P) after the indicated transfection. For all analyses, *P < 0.05 compared to the corresponding control.


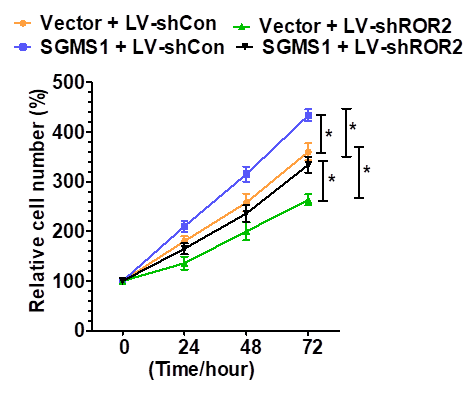


**Supplementary Figure S6.** DPSCs (3P) from young donors were transfected as indicated, and an MTS assay was performed to detect cell proliferation. * P < 0.05 compared to the corresponding control.


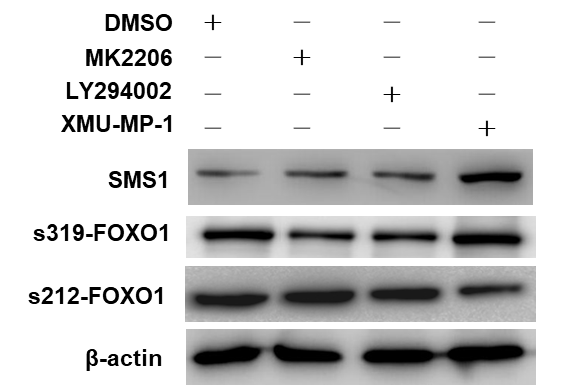


**Supplementary Figure S7.** DPSCs (12P) were treated with AKT pathway inhibitor (MK2206 or LY294002) or STK4 inhibitor (XMU-MP-1) for 2 h. Western blot analysis was performed to detect the SMS1, s319-FOXO1, and s212-FOXO1 protein expression; s319-FOXO1: Ser at position 319 of FOXO1, s212-FOXO1: Ser at position 212 of FOXO1.


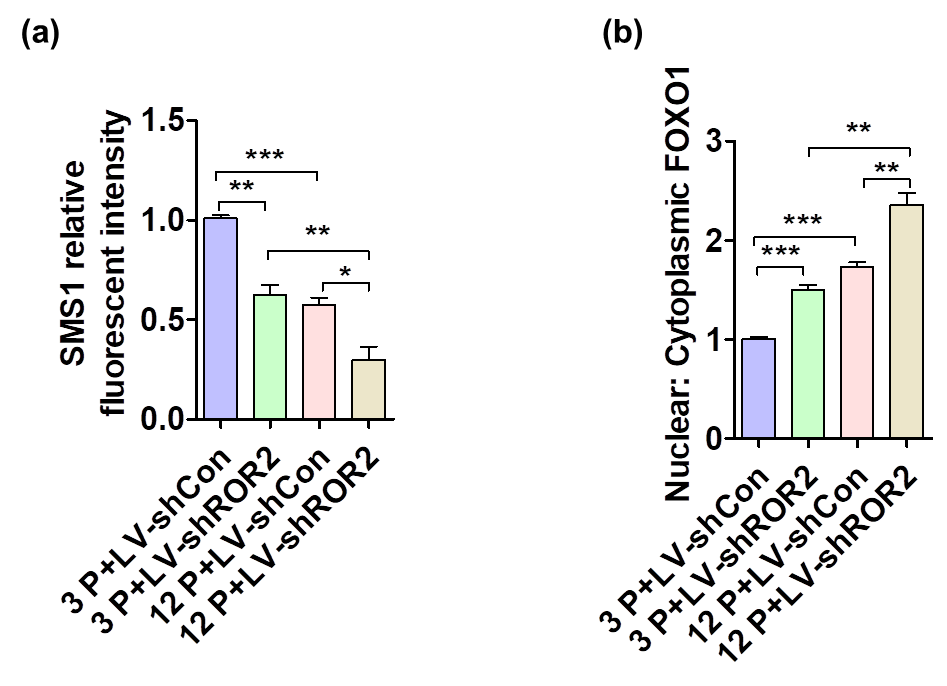


**Supplementary Figure S8. (a)** Comparison of the fluorescence intensity of SMS1 from Figure 6d. **(b)** Comparison of the ratio of fluorescence intensity of FOXO1 in the nucleus and cytoplasm from Figure 6d. For all analyses, * P < 0.05, ** P < 0.01, and *** P < 0.001 compared to the corresponding control.


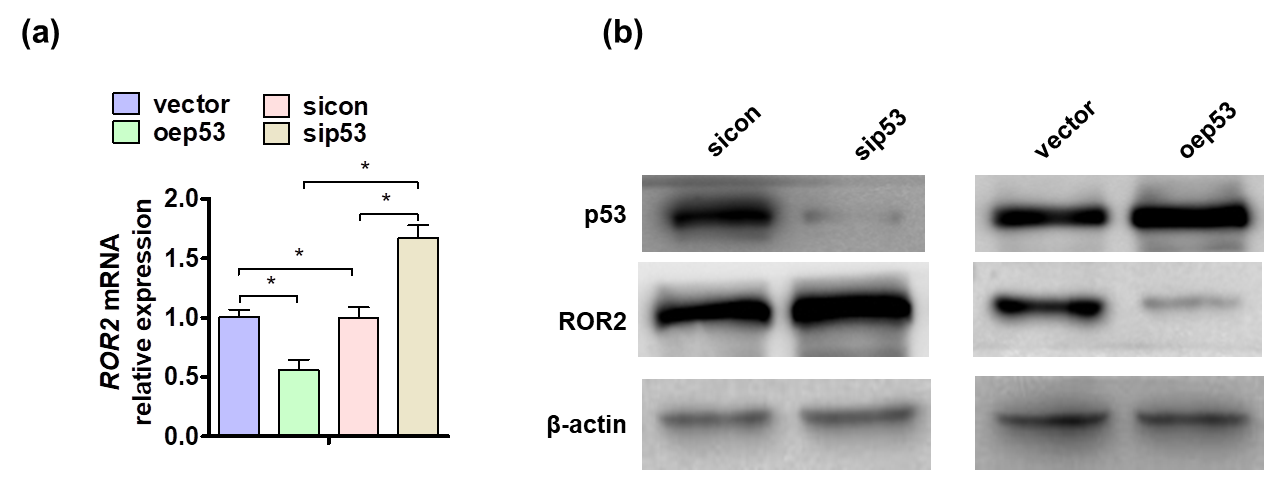


**Supplementary Figure S9. (a)** DPSCs from young donors (12P) were transfected with pcDNA3.1-p53 (oep53) or sip53 siRNA, or their control vector or siRNA, and RT-qPCR was performed to detect the ROR2 mRNA expression. * P < 0.05 compared to the corresponding control. **(b)** DPSCs were transfected as above and Western blot analysis was performed to estimate the p53 and ROR2 protein levels.
