# Supplementary material for: Downregulation of ROR2 promotes dental pulp stem cell senescence by inhibiting STK4‐FOXO1/SMS1 axis in sphingomyelin biosynthesis
Source: Aging Cell. 2021 Jul 18;20(8):e13430. doi: 10.1111/acel.13430 (PMC8373368; doi:10.1111/acel.13430)
Supplement: Supplementary file 2 — Table S1 [file ACEL-20-e13430-s001.pdf]

Supplementary table 1 Oligos used in the study

Name	Sequcence 5' to 3'
ROR2-F1	ACAGACTTCCCTGAGCTTGG
ROR2-R1	ATGTAAGGCATGGAGACCTGT
ROR1-F2	ATGTGGACTCCCTCCAGATG
ROR1-R2	GAAGACGAAGTGGCAGAAGG
FOXO1-F	GACCGAACAGGATGATCTTGGAGAAG
FOXO1-R	GCAAACGAGTAGCACGGCGTCTG
p16-F	CACCGAATAGTTACGGTCGGA
p16-R	CAATCGGGGATGTCTGAGGG
p21Cip1-F	ATACCGTGGGTGTCAAAGCA
p21Cip1-R	AGGGAGGGAGCCACAATACA
p53-F	AATCTACTGGGACGGAACAGCTTTGAGG
p53-R	GGAGAGGAGCTGGTGTTGTTGGG
SMPD1-F	CTGCGCACCCTCAGAATTGG
SMPD1-R	TGGTACACACGGTAACCAGG
SMPD2-F	GGAGGAAAAGGAACCGGGAG
SMPD2-R	AGGTTGAAGATCCGCAGTCG
SMPD3-F	GTGCTGAGTCTGAGGGAGG
SMPD3-R	GCCCTACTACATGGTGTC
SMPD4-F	TCCAGCAAAGGGTCCTGTGAA
SMPD4-R	GTGGAGGCCATAGGAAGCAA
SMS1-F	GAAAGCGTTCGCACCAGC
SMS1-R	GCCCATTCAGGGATCGTACA
SMS2-F	GGCAAGATGCTGTGGGATAGT
SMS2-R	TCTTCAACAGGTTCAGCGGG
DEGS1-F	CTTCCACTGAGCTGGAGTTTCC
DEGS1-R	TGAAATTGTGCCAGGGGAGC
SGPP1-F	TACTGCCTGTTCTGCTTCGG
SGPP1-R	ATAAGAGGGTACTGCCAGCG
ASAH1-F	ATCCAACCCGGTCACCTACC
ASAH1-R	CAGCAGGGAAGACAGTTGGT
SPTLC1-F	CTCCTCCCAGAGGAAGAACTGG
SPTLC1-R	TTGCTCTCTTTCAGGCCACT
CERS1-F	GACCCTCAGAGAACCCCCTA
CERS1-R	GGTGGGTCATGGAAGAAGGG
ARSG-F	ACCCCAACTTCCGTGGTTTT
ARSG-R	TGCTTGCACGCTGGATGA
Wnt5a-F	CTCCTCTCGCCCATGGAAT
Wnt5a-R	TGCAGTTCCACCTTCGATGT
CD73-F	CACTGCATTACAACCTGAAGTAGATAAG
CD73-F	GCCCATCATCAGAAGTGACTATG

CD105-F	GCGCTTGAACATCATCAGCC
CD105-R	AATCCCTCAGAGGCTTCACTG
CD34-F	CAGTCCCCCAACAGATGCTT
CD34-R	GCAGACACACCCAGCTAAGA
CD45-F	GGCAGTTGCTTGGATGATGC
CD45-R	GAAGGAAAATCATGCAACATTCTCC
BMP2-F	ACTCGAAATTCCCCGTGACC
BMP2-R	CCACTTCCACCACGAATCCA
RUNX2-F	CGCCTCACAAACAACCACAG
RUNX2-R	TCACTGTGCTGAAGAGGCTG
ALP-F	CAGACGTTCCATACCCCCAC
ALP-R	GGACCTTTGGCTCTCGACC
PPARG-F	CCAGAAGCCTGCATTTCTGC
PPARG-R	CACGGAGCTGATCCCAAAGT
AP2-F	GCTACACTGAGACTCCCGTC
AP2-R	GCGTGCGTGTTCCTTAATCC
CD44-F	CACACCCTCCCCTCATTCAC
CD44-R	TGGATGGCTGGTATGAGCTG
STK4-F	GACGGTACAGCTGAGGAACC
STK4-R	CAACAATCTGGCCGGTCTCT
ChIP1-F	TTCAAGGGGCCCAAACTGCGAC
ChIP1-R	CTTCCCGCGCGACGCGAC
ChIP2-F	CCGGGAGGCAGCCAGAGCAG
ChIP2-R	CACCGCTCGGCACCTGCCTC
ChIP3-F	CCCGCAGTTTCTGGTTCTGTAG
ChIP3-R	CTCTTCCCTGCAGCGCATACC
shROR2-F1	GCCCAAAUCAUAACUUUCAUU
shROR2-R1	UGAAAGUUAUGAUUUGGGCUU
shROR2-F2	GCGGUGGCUAAAGAAUGAUUU
shROR2-R2	AUCAUUCUUUAGCCACCGCUU
shROR1-F	GGCACUUCCAGUCACUUAUUU
shROR1-R	AUAAGUGACUGGAAGUGCCUU
si-STL4-F1	GCCCUCAUGUAGUCAAAUAUU
si-STL4-R1	UAUUUGACUACAUGAGGGCUU
si-STL4-F2	GCCAGAUUGUUGCUAUUAAUU
si-STL4-R2	UUAAUAGCAACAAUCUGGCUU
si-FOXO1-F1	CCCUCGAACUAGCUCAAAU UU
si-FOXO1-R1	AUUUGAGCUAGUUCGAGGGUU
si-FOXO1-F2	CGGGCUGGAAGAAUUCAAUUU
si-FOXO1-R2	AUUGAAUUCUUCCAGCCCGUU
